# Pathophysiological Role of Purines and Pyrimidines in Neurodevelopment: Unveiling New Pharmacological Approaches to Congenital Brain Diseases

**DOI:** 10.3389/fphar.2017.00941

**Published:** 2017-12-19

**Authors:** Marta Fumagalli, Davide Lecca, Maria P. Abbracchio, Stefania Ceruti

**Affiliations:** Laboratory of Molecular and Cellular Pharmacology of Purinergic Transmission, Department of Pharmacological and Biomolecular Sciences, Università degli Studi di Milano, Milan, Italy

**Keywords:** neurodevelopmental disorders, purine metabolism, enzyme deficiencies, adenosine, purine salvage pathway, P2X7 receptors

## Abstract

In recent years, a substantial body of evidence has emerged demonstrating that purine and pyrimidine synthesis and metabolism play major roles in controlling embryonic and fetal development and organogenesis. Dynamic and time-dependent changes in the expression of purine metabolizing enzymes (such as ectonucleotidases and adenosine deaminase) represent a key checkpoint for the correct sequential generation of the different signaling molecules, that in turn activate their specific membrane receptors. In neurodevelopment, Ca^2+^ release from radial glia mediated by P2Y_1_ purinergic receptors is fundamental to allow neuroblast migration along radial glia processes, and their correct positioning in the different layers of the developing neocortex. Moreover, ATP is involved in the development of synaptic transmission and contributes to the establishment of functional neuronal networks in the developing brain. Additionally, several purinergic receptors (spanning from adenosine to P2X and P2Y receptor subtypes) are differentially expressed by neural stem cells, depending on their maturation stage, and their activation tightly regulates cell proliferation and differentiation to either neurons or glial cells, as well as their correct colonization of the developing telencephalon. The purinergic control of neurodevelopment is not limited to prenatal life, but is maintained in postnatal life, when it plays fundamental roles in controlling oligodendrocyte maturation from precursors and their terminal differentiation to fully myelinating cells. Based on the above-mentioned and other literature evidence, it is now increasingly clear that any defect altering the tight regulation of purinergic transmission and of purine and pyrimidine metabolism during pre- and post-natal brain development may translate into functional deficits, which could be at the basis of severe pathologies characterized by mental retardation or other disturbances. This can occur either at the level of the recruitment and/or signaling of specific nucleotide or nucleoside receptors or through genetic alterations in key steps of the purine salvage pathway. In this review, we have provided a critical analysis of what is currently known on the pathophysiological role of purines and pyrimidines during brain development with the aim of unveiling new future strategies for pharmacological intervention in different neurodevelopmental disorders.

## Contribution of purinergic transmission to the development of the central nervous system

Neural development is a complex highly orchestrated process involving genetic, epigenetic, and environmental events that are crucial for shaping the architecture of the growing brain. Proliferation and migration of glia and neurons, followed by naturally occurring cell death of damaged or unnecessary cells, formation of synapses, myelination of axons, and generation of neuronal connections are indeed critically controlled by intrinsic and environmental factors at each stage during development (Stiles and Jernigan, 2010).

Purine and pyrimidine nucleotides are essential precursors for nucleic acid synthesis, but their functions are not limited to this. Purines act as metabolic signals, provide energy, control cell growth, are part of essential coenzymes, contribute to sugar transport and donate phosphate groups in phosphorylation reactions (Jankowski et al., 2005; Handford et al., 2006). Pyrimidines are involved in polysaccharide and phospholipid biosynthesis, detoxification processes, and in protein and lipid glycosylation (Lecca and Ceruti, 2008; Löffler et al., 2015). The nervous tissue produces huge amount of ATP, which is mainly employed to provide energy for membrane active pumps, such as Na^+^/K^+^ ATPase, and is fundamental to sustain synaptic transmission and the cooperation between neurons and glial cells (Bélanger et al., 2011; Micheli et al., 2011; Harris et al., 2012).

In the central nervous system (CNS), some purines serve more specialized roles, not only in neurons, but also in glial cells. Over the last 40 years it has been progressively understood that extracellular nucleotides exert many of their functions through the activation of ligand-gated P2X channels (the P2X1-7 subtypes) and of G protein-coupled P2Y receptors (the P2Y_1, 2, 4, 6, 11, 12, 13, 14_ subtypes; Abbracchio et al., 2006). Also, purine nucleosides exert receptor-mediated actions in all mammalian tissues and systems, including the brain. The vast majority of available data are focused on adenosine which activates 4 G protein-coupled receptors (the A_1, 2A, 2B, 3_ subtypes; Fredholm et al., 2011), collectively referred to as P1 receptors. An increasing body of evidence is now also pointing to specific effects of extracellular guanosine in modulating brain functions (for review, see Di Liberto et al., 2016). Nevertheless, the identification and cloning of guanosine receptor have failed so far. Overall, despite the presence of pyrimidine signaling molecules acting on the P2Y_2, 4, 6, 14_ receptor subtypes (von Kügelgen and Hoffmann, 2016) and on the P2Y-like receptor GPR17 (see section Involvement of the Purinergic System in Brain Alterations Observed in Down Syndrome), this system is referred to as the “purinergic system” (Burnstock, 2017). P1 and P2 receptors are widely expressed throughout the body, where they exert a variety of physiological functions, including neurotransmission (Burnstock, 2017). Some specific subtypes are also crucially involved in controlling brain development.

### P2Y receptors in CNS development

The first hints of a specific role for purinergic receptor signaling during development came from studies in *Xenopus*, where ATP degradation to ADP by E-NTPDase-2 in the anterior neural plate, with subsequent activation of the P2Y_1_ receptor subtype, leads to the induction of *Pax6, Rx1*, and *Six3* genes. These genes encode for transcription factors collectively referred to as eye field transcription factors (EFTF), and are fundamental to promote and sustain eye development (Massè et al., 2007). Altering purinergic signaling, by either overexpressing or downregulating some of the molecular components of this pathway, leads to abnormal eye development, thus confirming the crucial role of extracellular nucleotides during tissue generation and cell specification in embryos.

**Figure 1:**
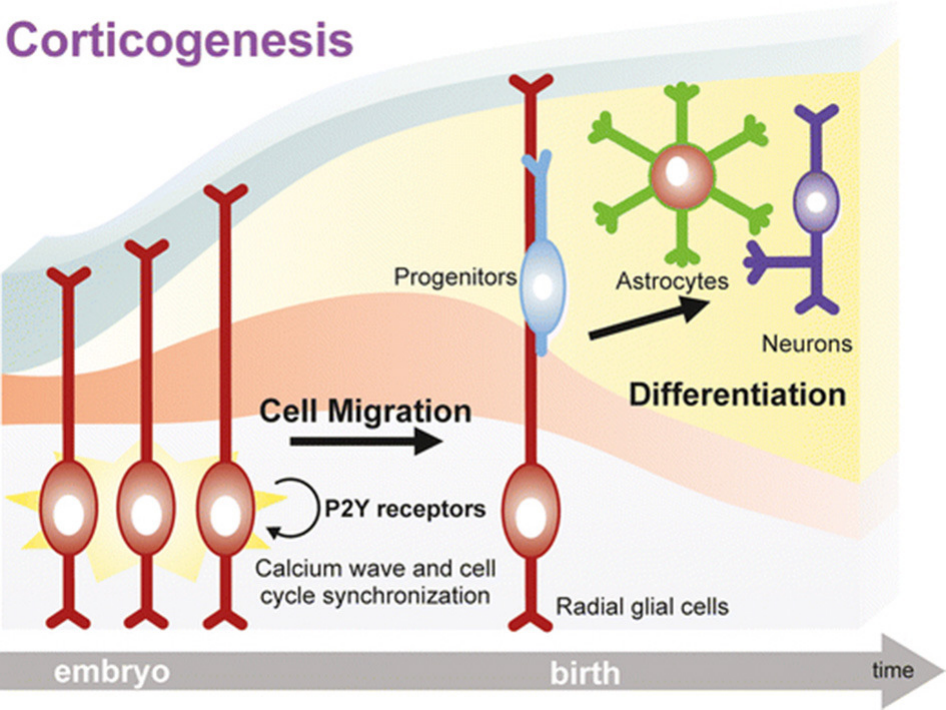
A key role for purinergic receptors in cell cycle synchronization and progenitor migration during CNS development. Radial glia cells originate from neuroepithelial cells after the onset of neurogenesis, and they bear two essential functions: (i) act as neural progenitor cells, and (ii) provide a scaffold for migration and organization of the cortex structure. Following activation of purinergic receptors, mostly P2Y subtypes, calcium waves sustain cell cycle progression of migrating precursors, which are synchronized with neighboring radial glial cells thanks to a sustained paracrine ATP release. Progenitors eventually mature into neurons and astrocytes organizing the cortical structure. Neurogenesis and gliogenesis initiated by purinergic signaling in the embryonic brain continue during postnatal development. Reproduced from Ulrich et al. (2012) with permission (license # 4213050265596) from Springer.

Data have been further confirmed and expanded to the mammalian brain, where it has been demonstrated that the expression of specific P2Y receptor subtypes is plastic and tightly controlled at different embryonic stages, and correlates with their recruitment in controlling brain development (Oliveira et al., 2016). For example, P2Y_1_ receptor-mediated calcium signaling is fundamental for the specification of the cortical layers. At this stage of development, daughter cells derived from neural precursors located in the ventricular/subventricular zone start their migration toward their final localization in the developing cortex where they differentiate to either neurons or astrocytes (Pino et al., 2017). Radial glial cells, a peculiar type of glial cells, act as both neuronal progenitors in the ventricular/subventricular zone through their asymmetric division and as “guiding sign” for migrating cells. In fact, their long radial process extends up to the subpial surface, and constitute a “highway” for migrating cells to find their final localization in the correct cortical layer (Ulrich et al., 2012; Figure 1).

Extracellular nucleotides control both radial glial cell proliferation and migration of developing neuroblasts and glial cells. In fact, a paracrine ATP signaling is established through its degradation to ADP and activation of P2Y_1_ receptors, leading to the generation of [Ca^2+^]_i_ waves that propagate through hemichannel and gap junctions, thereby synchronizing cell cycle and migration (Weissman et al., 2004; Lecca et al., 2016; Figure 1). In turn, calcium waves amplify ATP release accompanied by other neurotransmitters and growth factors, which further contribute to drive neural precursor migration toward developing cortical layers, where they terminally differentiate to neurons or glial cells (Ulrich et al., 2012).

Furthermore, extracellular ATP acts as one of the main activity-dependent axonal signal and activates P2 receptors on oligodendrocyte precursors cells (OPCs), which generate mature oligodendrocytes during early post-natal development (Fields and Stevens, 2000). ATP and uracil nucleotides interact with growth factors to trigger specific intracellular pathways that regulate OPC proliferation, migration, terminal maturation, and myelination (Fumagalli et al., 2016).

Neurotransmitters and growth factors released by migrating cells and radial glia are also fundamental for driving neuronal differentiation and maturation, and for neurite extension and myelination. As recently elegantly reviewed (Heine et al., 2016), the purinergic system is crucially involved also in these later stages of neuronal development, thanks to the recruitment of several receptor subtypes, activated by either nucleotides or adenosine. For example, both purine P2Y_1, 13_ and pyrimidine P2Y_2_ receptor subtypes have been positively correlated to neurite elongation, with the latter specifically linked to the α_V_ integrin/Rho/ROCK pathway (del Puerto et al., 2012; Peterson et al., 2013). A significant contribution to these events is also provided by astrocytes, and the purinergic system is also directly involved in controlling their functions (Buffo et al., 2010; Heine et al., 2016).

Neurogenesis and gliogenesis initiated by purinergic signaling in the embryonic brain continue during postnatal development, and the ventricular/subventricular zone is recognized in the adult brain as neurogenic niche, together with the subgranular layer of the hippocampus (Boda et al., 2017). *In vitro* and *in vivo* studies have demonstrated that the ADP-responsive P2Y_1_ receptor subtype endures in its role of modulator of precursor cell proliferation and differentiation throughout life (Suyama et al., 2012; Boccazzi et al., 2014). Several other P2Y (and also P2X) receptor subtypes have been found expressed in the adult neurogenic niches (Mishra et al., 2006; Stafford et al., 2007; Grimm et al., 2009), and could therefore contribute to modulate neural precursor cell proliferation, differentiation, and recruitment following brain injury, in an often-unsatisfactory attempt to damage repair.

### The elusive role of P2X7 receptor in controlling neuronal functions

**Figure 2:**
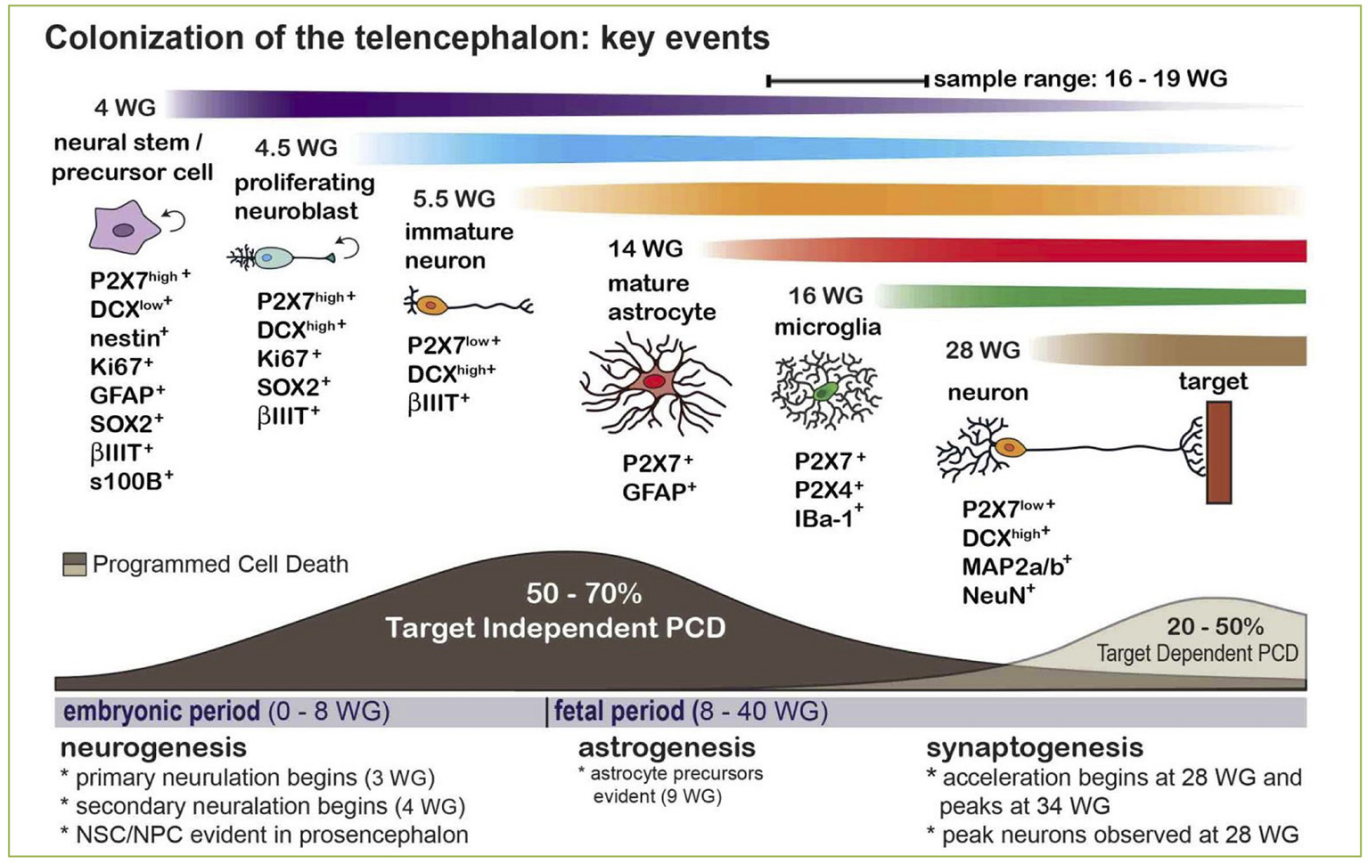
Expression and phagocytic roles for the P2X7 receptor in the developing human CNS and its contribution to Programmed Cell Death (PCD). The scheme indicates that naturally occurring PCD is fundamental in CNS development, due to cell overproduction. It can be divided into: (i) target-independent PCD of proliferating neural stem/precursor cells or neuroblasts, and (ii) target-dependent PCD of postmitotic neurons after establishing synaptic contact with their targets. The phagocytosis and elimination of dead precursors (~50–70% is eliminated by target-independent PCD prior to maturation) is mediated via the scavenger receptor P2X7 expressed by surrounding neurobalsts. The colonization of the developing CNS parenchyma by professional phagocytes (i.e., microglia/macrophages) or the full maturation of astrocytes occurs after 14–15 weeks of gestation (WG). Reproduced from Gu et al. (2015) under a Creative Commons (CC) license.

Although for many years the expression of P2X7 receptor in adult brain has been confined to glial cells (i.e., astrocytes and microglia), a lively debate is currently open on its expression by adult neurons, with arguments both in favor and against this statement (Illes et al., 2017; Miras-Portugal et al., 2017). What is now widely accepted is that P2X7 is highly expressed by neuroblasts and neural precursors during brain development, where it inhibits cell proliferation and neurite outgrowth, but promotes neuronal differentiation (Heine et al., 2016; Oliveira et al., 2016). Interestingly, recent data point for a new role of P2X7^+^ neuroblasts which also express high levels of doublecortin, the typical marker of developing neurons (Boda et al., 2017). These cells are in fact endowed with the peculiar ability to phagocyte surrounding cells dying by programmed cell death well before brain colonization by specialized phagocytes, such as microglia/macrophages (Gu et al., 2015; Lovelace et al., 2015; Figure 2). Based on these data, since programmed cell death is a fundamental process during brain development and shaping, leading to the elimination of unnecessary, damaged, or exceeding cells, a new fundamental role for P2X7-mediated purinergic signaling during embryonal life is now emerging.

### Contribution of adenosine P1 receptors to neurodevelopment and neuromodulation

Adenosine has been shown to dramatically affect embryonic development as well. In fact, it is directly involved in the elimination of interdigital membranes, in the apoptotic death of clones of autoreactive lymphocytes in the thymus, and in the morphogenetic outgrowth of vertebrate limb buds (Jacobson et al., 1999). Moreover, the adenosine catabolizing enzyme adenosine deaminase (ADA) is expressed at high levels in the placenta, and its pharmacological inhibition disrupts fetal development (Knudsen et al., 1992), thus suggesting that developing tissues and organs are highly sensitive to increased adenosine concentrations. A direct link with the activation of specific P1 receptor subtypes has not been demonstrated; it is more likely that non-receptor mediated effects are involved in this pro-apoptotic and toxic actions of adenosine, as already demonstrated in ADA deficiency and in other disorders (see below; Jacobson et al., 1999). Additionally, in the developing brain the expression of adenosine kinase, the enzyme responsible for the rephosphorylation of adenosine to nucleotides, is switched from neurons during life *in utero* to exclusive astrocytic expression along with progressive brain maturation (Studer et al., 2006). It is therefore clear that a tight control of adenosine levels is fundamental for brain development and neural plasticity (Boison et al., 2012). A role for adenosine in promoting neurite outgrowth through the A_2A_ receptor subtype (Heine et al., 2016), and in fostering the differentiation of OPCs and their ability of myelinate axons (Butt et al., 2014) has been also highlighted.

Adenosine is involved in the regulation of several signaling pathways in CNS (Boison, 2008), acting as a neuromodulator through multiple mechanisms, including the control of neurotransmitter release, or via regulatory effects on glial cells (Boison et al., 2010, 2012). As extensively reviewed elsewhere (Fredholm et al., 2005), activation of A_1_ receptors inhibits the release of neurotrasmitters, such as dopamine and glutamate, and decreases neural excitability by inducing post-synaptic hyperpolarization. Conversely, A_2A_ receptors promote neurotransmitter release. Based on these interactions with glutamatergic and dopaminergic neurotransmission, a role for adenosine in neurodevelopmental defects at the basis of schizophrenia has been proposed (Lara and Souza, 2000; Lewis and Levitt, 2002; see section The Adenosine Dysfunction Hypothesis in Neurodevelopment). A role for A_2B_ and A_3_ adenosine receptors (both expressed in brain) in neural development still remains to be unveiled.

## Neurodevelopmental disorders associated to alterations in purine and pyrimidine metabolism

### Defects in nucleotide metabolism

**Figure 3:**
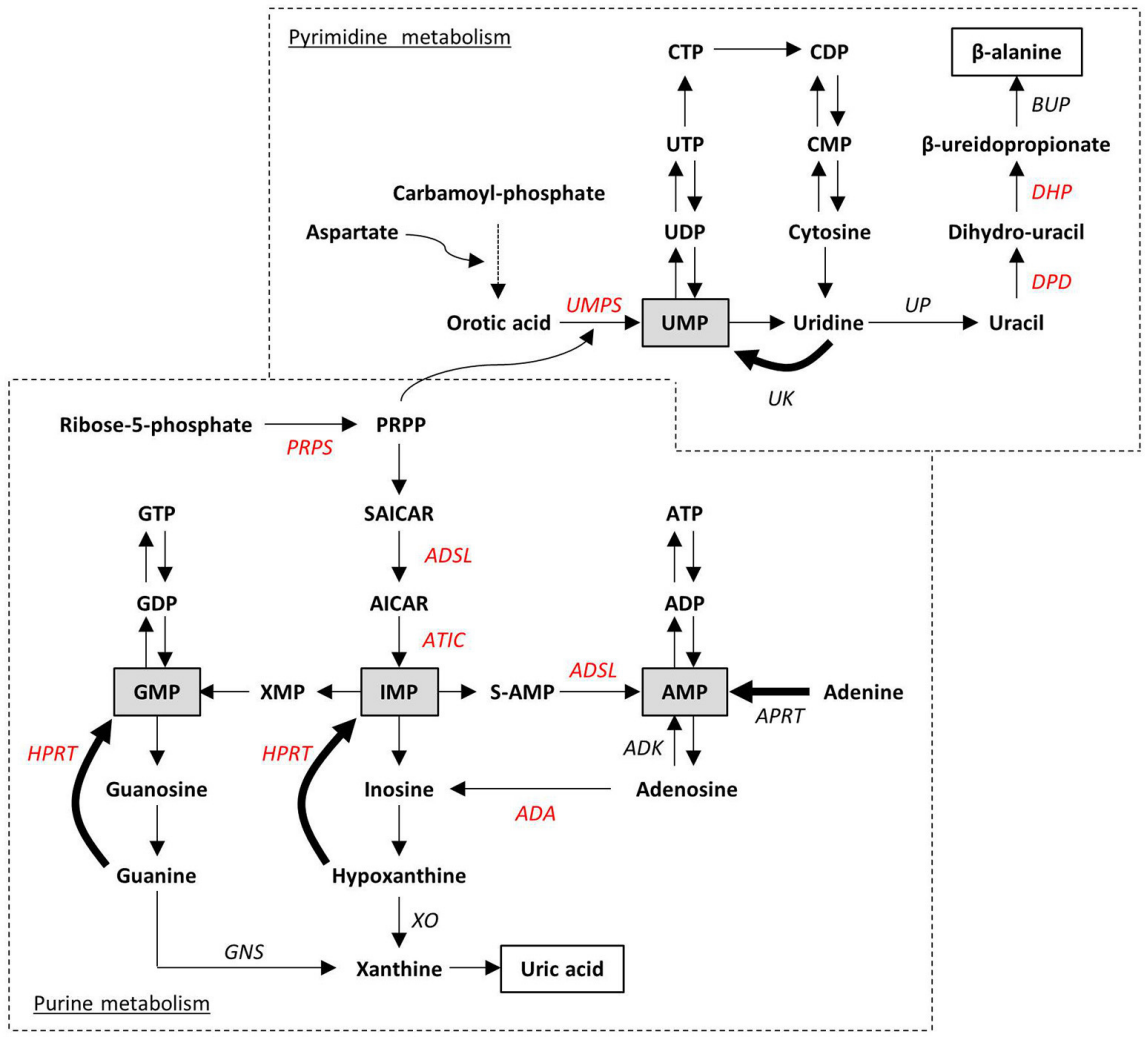
Schematic representation of purine and pyrimidine metabolism. Ribose-5-phosphate and carbamoyl-phosphate are the starting points of the two *de novo* biosynthesis pathways. Salvage pathways are indicated with bold arrows. Monophosphate intermediates, representing the link between *de novo* biosynthesis and salvage pathways, are highlighted in gray boxes. End-products of purine and pyrimidine catabolism (i.e., uric acid and β-alanine) are in white boxes. Impairment at any enzymatic step can lead to inborn disorders. Enzymes whose altered functions have already been associated to the neurodevelopmental disorders described in the text are indicated in red. ADA, Adenosine deaminase; ADK, Adenosine kinase; ADSL, adenylosuccinate lyase; APRT, adenine phosphorybosyl-transferase; ATIC, AICA-ribotidetransformylase/IMP cyclohydrolase; BUP, β-ureidopropionase; DHP, dihydropyrimidinase; DPD, dihydropyrimidine dehydrogenase; GNS, guanase; HPRT, hypoxanthine-guanine phosphorybosyl-transferase; PRPP, 5-phosphorybosyl-1-pyrophosphate; PRPS, PRPP synthetase; S-AMP, adenylosuccinate; SAICAR: succinylaminoimidazole carboxamide ribotide; XO, xanthine oxydase; UK, uridine kinase; UMPS, UMP synthetase; UP, uridine phosphorylase.

Purine *de novo* biosynthesis is a complex, energy-expensive process. As depicted in Figure 3, it begins with the formation of phosphoribosyl pyrophosphate (PRPP) and leads to the first fully formed nucleotide, inosine 5′-monophosphate (IMP), which can be subsequently converted into either AMP or GMP (Kelley and Andersson, 2014). PRPP is also utilized to convert the nucleobase orotic acid into uridine monophosphate (UMP), the first step of the biosynthesis of pyrimidines. Alternatively, intracellular nucleosides coming from the diet or from the dephosphorylation of endogenous nucleotides can be recycled by the salvage pathway. The catabolism of residual free bases generates uric acid in case of purines, and β-alanine and β-aminoisobutyric acid in case of pyrimidines to be excreted as end products. Both *de novo* and salvage pathways are feedback-regulated by their end products, whereas purine catabolism is mostly regulated by substrate availability (Micheli et al., 2011). Only 10–30% of the free purines generated by intracellular metabolism are degraded or excreted (Torres and Puig, 2007), thus highlighting the importance of nucleotide salvage, which is fundamental in brain tissues; in addition, salvaged IMP from the liver is transported by erythrocytes to the brain and other tissues, where it is released for conversion to ATP or GTP (Ipata et al., 2011).

Considering the crucial roles of purines, pyrimidines, and their derivatives in brain development (see section Contribution of Purinergic Transmission to the Development of the Central Nervous System), it is evident that alterations in their synthesis, catabolism, and concentrations may lead to significant functional consequences. Inborn errors in purine metabolism are usually rare, and characterized by the absence or abnormal concentrations of purine nucleotides in cells, or by the presence of toxic intermediates in body fluids. At present, more than 35 enzyme defects of nucleotide synthesis, salvage, and catabolism of both purines and pyrimidines have been identified, some of which are associated with serious clinical consequences during development (Table 1; for review see Micheli et al., 2011). These disorders, previously considered as pediatric diseases, are now increasingly recognized in adults with milder phenotypes. Although purines and pyrimidines are essential in all tissues, the clinical outcomes of these disorders often suggest that the CNS is more seriously affected than other organs.

**Table 1:** Description of the most relevant pathologies where an involvement of purines and pyrimidines in neurodevelopmental alterations has been hypothesized or demonstrated.

**Disease**	**Causes and incidence**	**Symptoms**	**References**
ADA deficiency (ADA-SCID)	Autosomal recessive mutation in the ADA gene (20q13.12). Estimated incidence 1:200,000-1,000,000.	Severe immunodeficiency Seizures Autistic behaviors	Bottini et al., 2001; Micheli et al., 2011
ADSL deficiency	Autosomal recessive mutation in the ADSL gene (22q13.1). Enzyme deficiency leads to accumulation of toxic derivatives of adenosine in body fluids. Rare disorder (80 patients worldwide).	Developmental delay Seizures Hypotonia Autistic features Brain atrophy	Micheli et al., 2011; Jinnah et al., 2013
ATIC deficiency	Autosomal recessive mutation in the ATIC gene (2q35). Accumulation of toxic derivatives of adenosine in body fluids. One single case with complete deficiency of the ATIC enzyme was described.	Intellectual disabilities Blindness Epilepsy	Marie et al., 2004
Autism spectrum disorder	Still to be clarified. The concurrence of genetic and environmental factors has been suggested. It manifests in the first 36 months of life. Estimated incidence 30-60:10,000.	Impairments in social communication and interactions Stereotyped repetitive behaviors Affective instability	Voineagu et al., 2011; Lauritsen, 2013
Dihydropyrimidinase (DHP) deficiency	Autosomal recessive mutation in the DPYS gene (8q22). 11 cases with complete DHP absence have been described. Heterozygous subjects are asymptomatic and show some of the described symptoms upon 5-FU administration (pharmacogenetic syndrome).	Developmental delay Epilepsy	van Kuilenburg et al., 2003, 2010; Micheli et al., 2011
Dihydropyrimidine dehydrogenase (DPD) deficiency	Autosomal recessive mutation in the DPYD gene (1p21.3). Full or partial DPD deficiency is estimated to be present in about 3%-5% of the population. Most of them are asymptomatic. Some of the severe outcomes were observed upon administration of 5-FU (pharmacogenetic syndrome).	Seizures Motor and mental retardation Autistic features Gastroenteric disorders Myelosuppression, myelopathy	van Kuilenburg et al., 2003
Down Syndrome (DS)	Trisomy of chromosome 21 leading to the aberrant overexpression of genes and miRNAs. Incidence: 1:700, 1:1,000 live births.	Broad clinical spectrum Platelet disorders Cardiac alterations Mental retardation Accelerated aging with early deposition of β-amyloid-plaques and Alzheimer's-like features	Dierssen, 2012
Hereditary orotic aciduria (UMPS deficiency)	Autosomal recessive mutation in the UMPS gene (3q21). Rare disorder (20 cases worldwide).	Orotic aciduria, crystalluria, Megaloblastic anemia, immunodeficiency Developmental delay Motor impairment, hypotonia	Wortmann et al., 2017
Hypophosphatasia	Hypomorphic mutations in the *ALPL* gene, encoding TNAP Estimated incidence: 1/100.000.	Rickets Osteomalacia Seizures	Whyte, 2010; Sebastián-Serrano et al., 2016
Lesch-Nyhan syndrome	X-linked mutation in the HPRT1 gene (Xq26.2-q26.3), producing a defective form of the enzyme. Uric acid precipitates in the body fluids. Alteration in dopamine levels, in particular in basal ganglia. Estimated incidence 1:500,000 (affects only males).	Hyperuricemia, nephrolithiasis, gout Dystonia, choreoathetosis, extrapyramidal symptoms Intellectual disability, self-injurious behavior	Wong et al., 1996; Ceballos-Picot et al., 2009; Jinnah et al., 2013
PRPP synthetase 1 (PRPS1)-deficiency	X-linked point mutation in the PRPS1 gene, with partial or complete loss of enzymatic activity. The most severe outcome is Arts syndrome. Extremely rare.	Developmental delay Hypotonia, ataxia, hearing impairment, optic atrophy, peripheral neuropathy Premature death due to recurrent infections	Duley et al., 2011
PRPS1-superactivation	X-linked point mutation in the PRPS1 gene (Xq22.4), leading to increased enzymatic activity in cells with high RNA/protein turnover. Conversely, the mutated enzyme is unstable in neural cells and erythrocytes, where very low residual activity was found. Rare disorder (30 families worldwide).	Cognitive impairment Ataxia Hypotonia Sensorineural deafness Hyperuricemia, gout, kidney failure	Duley et al., 2011
Schizophrenia	Chronic and severe mental disorder with a typical onset in late adolescence or early adulthood. Caused by a combination of genetic susceptibility and environmental perturbations. Estimated incidence: 1.5:10,000.	Positive symptoms: visual or auditory hallucinations Negative symptoms: apathy, anhedonia and alogia Cognitive symptoms: altered ability to think clearly and to sustain attention	Lewis and Levitt, 2002; McGrath et al., 2008

In the following paragraphs, we will introduce some of these disorders, focusing on the possible links between enzyme alterations during development and their neurological outcomes.

#### Defects in purine *de novo* biosynthesis and catabolism

PRPP synthetases (PRPS) catalyze PRPP synthesis from Mg-ATP and ribose-5-phosphate, and represent the first step in purine synthesis. Modulation of PRPS1 activity by its substrates, inhibitors (ADP and GDP), activators (Mg^2+^ and organic phosphate), and end-products determines the intracellular levels of PRPP, an essential cofactor for both *de novo* biosynthesis and salvage pathway (see section Defects in Nucleotide Metabolism; Figure 3; Micheli et al., 2011). Thus, defects in PRPS have serious consequences for several essential processes, such as nucleic acid synthesis, cellular metabolism, and signaling (de Brouwer et al., 2010).

Three isoforms of PRPS have been identified in humans, two of which are expressed in the brain: PRPS1 and PRPS2. In particular, altered activity of PRPS1 was associated to several distinct diseases. The most characterized is enzyme superactivity resulting from point mutations affecting the allosteric regions that regulate the enzyme switching off. Purine overproduction leads to hyperuricemia, and patients often show increased hypoxanthine and xanthine levels in cerebrospinal fluid (Torres et al., 2016). However, low purine nucleotide, in particular GTP, levels are observed, since the deregulated enzyme is also unstable and becomes inactive in anucleated or post-mitotic cells, such as erythrocytes and brain cells, in which the enzyme turnover is low or absent. Neurological outcomes include mental retardation, early-onset hypotonia, ataxia, delayed motor development, sensorineural deafness, and optic atrophy (de Brouwer et al., 2010). Interestingly, similar neurological symptoms are observed in patients affected by Arts syndrome, a severe disease in which a missense mutation completely inactivates PRPS1 (Duley et al., 2011). Other mutations in the same enzyme are associated to sensorineural symptoms (e.g., neuropathy, deafness) not directly related to specific defects in neurodevelopment. There is no univocal explanation of the variety of neurological symptoms, but increased oxypurine production and GTP depletion in the CNS could play an important role (de Brouwer et al., 2010).

The last steps of purine *de novo* biosynthesis (see Figure 3) require the sequential activity of two enzymes: adenosylosuccinate lyase (ADSL) and AICAR transformylase/IMP cyclohydrolase (ATIC). ADSL catalyzes both the conversion of succinylaminoimidazole carboxamide ribotide (SAICA-R) into AICA-ribotide (AICAR), and of adenylosuccinate (S-AMP) to AMP. Altered function of ADSL leads to the accumulation of succinyl-purines in body fluids, mainly in cerebrospinal fluid and urines, with consequent neurotoxic effects (Jinnah et al., 2013). The clinical manifestations of mutations in this enzyme are very diverse and include psychomotor retardation, seizures, and autistic features. The ratio between S-adenosine (a toxic intermediate produced by dephosphorylation of S-AMP) and SAICAR is inversely proportional to the onset and severity of symptoms.

ATIC deficiency, described in one single case, also leads to the formation of toxic intermediates, mainly AICAR and its ribosides (called ZMP, ZDP, and ZTP), with mental retardation, epilepsy, brachycephaly, dysmorphic features, and blindness (Marie et al., 2004). In this patient, erytrocyte ATP and AMP concentration was lowered by 60%, but no significant changes in other nucleotides were observed. AICAR is neurotoxic in undifferentiated neuroblastoma cells and triggers apoptosis, and shows a marked inhibition of carbohydrate and lipid metabolism in the liver through the activation of AMP-dependent protein kinase (AMPK; Garcia-Gil et al., 2006). ATIC deficiency and consequent depletion of purines could be particularly relevant during embryonic development and organogenesis, when *de novo* synthesis is more important. This consideration may be true for several other defects in purine and pyrimidine metabolism in which the link with some neurological features are not fully clarified.

Dysfunction in ADA, the enzyme catalyzing the deamination of adenosine to inosine (see section Contribution of Purinergic Transmission to the Development of the Central Nervous System), is correlated to a severe combined immunodeficiency (named ADA-SCID). Accumulation of adenosine affects physiological methylation reactions, induces apoptosis of thymic lymphocytes and inhibits ribonucleotide reductase, thus interfering with DNA synthesis and repair (Joachims et al., 2008); these effects could explain the immunological impairment observed in ADA-deficient patients. Neurological symptoms, such as seizures and autistic behaviors, usually absent in early infancy and increasing in severity with age, were also described. Interestingly, reduced ADA activity has been reported in the serum of autistic children, in association with a polymorphism in the ADA gene (Bottini et al., 2001). *In vivo* studies in animal models support the hypothesis of adenosine toxicity during embryonal life, similarly to what observed in lymphocytes (see above). Both receptor-mediated and receptor-independent mechanisms have been demonstrated to be at the basis of adenosine toxicity (Jacobson et al., 1999). When specific receptor subtypes are involved, high adenosine levels may either overactivate or inhibit cAMP signaling, thus starting a cascade of events that leads to impaired neurotransmission (see also section The Adenosine Dysfunction Hypothesis in Neurodevelopment).

These examples of alterations in purine biosynthesis and catabolism clearly highlight how the blockade of the same pathway can have distinct neurological outcomes. In some of these disorders the concentration of purine nucleotides in body fluids is only slightly changed, probably due to supply by the salvage pathway.

#### Deficiencies in the purine salvage pathway

The hypoxantine-guanine phosphoribosyl transferase (HPRT) enzyme catalyzes the reutilization of hypoxantine and guanine in the energetically favorable synthesis of the nucleotides IMP and GMP, respectively, (see bold arrows in Figure 3). Any mutation in the human *HPRT1* gene, located on the X chromosome, produces defective forms of the enzyme that are unable to recycle nucleotides; thus, accelerated *de novo* biosynthesis is stimulated as a result of the accumulation of PRPP and reduced inhibition by end-products (Deutsch et al., 2005). This unbalance causes overproduction of uric acid that can precipitate in body fluids with subsequent hyperuricemia, nephrolithiasis, and gout. The most severe disorder associated with a very low residual HPRT activity is Lesch-Nyhan syndrome, also characterized by motor impairment, with dystonia and extrapyramidal symptoms, intellectual disabilities, and dramatic compulsive self-mutilation such as biting digits, lips, or buccal mucosa (Nguyen and Nyhan, 2016). Treatment with allopurinol, an inhibitor of xanthine oxidase, reduces plasma concentrations of uric acid, with no effect on neurological symptoms (Deutsch et al., 2005), suggesting that in the brain more complex metabolic mechanisms are involved.

Although the pathogenesis of neurological and neurobehavioral manifestations is not clearly understood, several pieces of evidence support the idea that HRPT deficiency strongly influences early development of dopaminergic neurons (Ceballos-Picot et al., 2009; Kang et al., 2011). Post mortem brains from Lesch-Nyhan subjects did not show any morphological abnormality or signs of degeneration (Del Bigio and Halliday, 2007; Göttle et al., 2014). However, voxel-based morphometry imaging revealed a significant reduction of brain volumes in Lesch-Nyhan patients, likely due to developmental deficits, in particular in clusters of white matter including the nigrostriatal dopamine pathway (Schretlen et al., 2015). Moreover, positron emission tomography and autopsy studies showed a 60–90% reduction in both dopamine levels and dopamine uptake in basal ganglia of patients (Wong et al., 1996). In accordance, neurochemical analysis revealed alterations of brain neurotransmitters, with decreased dopaminergic synapses in the striatum (Visser et al., 2000). All these changes were consistent with the extrapyramidal symptoms of the syndrome.

Several authors have tried to explain the molecular link between the altered purine metabolism and neuronal development. It has been recently hypothesized that the excess in hypoxantine concentrations is the trigger for subsequent neurochemical abnormalities (Torres et al., 2016). Indeed, normal neurons are very efficient in consuming almost all the hypoxanthine synthesized from nucleotide catabolism and they release virtually no hypoxanthine into the culture medium. Due to feedback enzyme regulation, defective salvage is counterbalanced by an increased *de novo* purine synthesis. HPRT-deficient rat neuroblastoma cell line B103, used as an experimental *in vitro* model of the disease, showed normal excretion of xanthine, but hypoxanthine is not recycled to IMP, leading to a 15-fold increase in extracellular hypoxanthine excretion (Pelled et al., 1999). These abnormal concentrations of hypoxantine, in turn, diminish adenosine uptake into the cells by a competitive mechanism through equilibrative nucleoside transporters (Prior et al., 2007) and increase extracellular adenosine concentrations, thus prolonging the activation of its receptors. As mentioned above, one of the main effects of adenosine in the CNS is the inhibition of neurotransmitter release through the A_1_ receptor subtype, with decreased neural excitability by post-synaptic hyperpolarization. However, adenosine can also induce opposite effects through the A_2A_ receptor (Fredholm et al., 2005). These receptor subtypes are expressed in different areas of the developing brain, and their timely recruitment regulates a correct integration between excitatory and inhibitory processes. It is likely that in Lesch-Nyhan syndrome excitatory A_2A_-mediated signaling is predominant compared to A_1_-mediated inhibitory effects. Thus, alterations in adenosine levels generate an imbalance in neurotransmission, that could be responsible for some of the symptoms observed in patients, including aggressive and self-injurious behaviors. Moreover, in post mitotic neurons hypoxanthine also increases the expression of the adenosine A_2A_, dopamine D1, and serotonin 5-HT_7_ receptors further potentiating their signaling (Torres and Puig, 2015).

#### Neurodevelopmental diseases and pyrimidine metabolism

Ten defects in pyrimidine metabolism have been described, but their incidence is probably underestimated (Balasubramaniam et al., 2014). As for purines, pyrimidine-based compounds are ubiquitous, and this may explain the clinical heterogeneity of the symptoms of the deficiency of specific enzymes. Clinical manifestations include unexplained anemia, delayed development, seizures, neonatal fitting, microcephaly, mental retardation, and dysmorphic features.

The typical disorder of pyrimidine metabolism is orotic aciduria, known as a megaloblastic anemia accompanied by renal impairment, due to a deficiency in uridine monophosphate synthetase (UMPS; see Figure 3), a bifunctional enzyme in the *de novo* pyrimidine synthesis. The first reaction catalyzes the conversion of orotate to orotidine monophosphate, thanks to orotate phosphoribosyltransferase (OPRT) activity. In the second step, orotidine decarboxylase (ODC) generates UMP. Very high orotate levels (up to 400-fold compared to control subjects) are found in urine and plasma of patients suffering from the disease (Wortmann et al., 2017). Children with UMPS deficiency have a strong pyrimidine starvation and, if untreated, can develop growth retardation, intellectual disability, and epilepsy.

Interestingly, pyrimidine nucleotide starvation can be partially rescued by exogenous administration of uridine (see section Toward a Purinergic-Based Therapy for Congenital Neurodevelopmental Disorders?). Indeed, dietary pyrimidines (mainly cytosine and uracil derivatives) are able to pass into the circulation, where they are salvaged starting from their nucleosides (cytidine and uridine, respectively). Conversely, most purines do not enter the bloodstream as they are converted to uric acid during their transit across the small intestine (Duley et al., 2011). Uridine has been utilized in clinics as an anticonvulsant in the treatment of autism-associated seizures (Kovács et al., 2014).

A small number of patients show specific enzymatic deficiencies in the catabolic pathways for pyrimidines, mostly showing with mental retardation, seizures, or both. In this case, toxic accumulation of pyrimidines and their metabolites were found. Dihydropirimidine dehydrogenase (DPD) catalyzes the first step of the catabolism of pyrimidine bases, i.e., the reduction of uracil and thymine to dihydrouracil and dihydrothymine, respectively. Point mutations in the DPYD gene lead to a defective enzyme that, in homozygous subjects, results in thymine-uraciluria. Complete DPD-deficiency is associated to convulsions, motor, and mental retardation in the most severe forms, with autistic features and NMR evidence of severe delay in brain myelination (Enns et al., 2004). Usually, psychomotor development is normal at birth, but progressively worsens during the first years of life with speech retardation and various degrees of developmental delay (van Kuilenburg et al., 2010). Heterozygous subjects are usually asymptomatic, but can show gastroenteric disorders, myelosuppression, cerebellar ataxia, mental deterioration, and myelopathy if treated with the DPD substrate 5-fluorouracil (5-FU), a pyrimidine analog widely used as antineoplastic drug. In fact, enzyme deficiency prevents the degradation of 5-FU, whose accumulation quickly reaches toxic concentrations (van Kuilenburg et al., 2003).

The second enzyme of pyrimidine catabolism is dihydropirimidinase (DHP), responsible for the reversible hydrolysis of dihydrouracil and dihydrothymine coming from the previous step to N-carbamyl-β-alanine and N-carbamyl-β-aminoisobutyric acid, respectively. DHP is expressed in liver and kidney, whereas no activity was found in brain. However, DHP-deficient subjects can show strong neurological manifestations similar to those observed in DPD deficiency, including the pharmacogenetic syndrome induced by 5-FU. In fact, toxic accumulation of dihydropyrimidines was found not only in blood, but also in cerebrospinal fluid, thus confirming that these metabolites cross the blood-brain barrier (van Kuilenburg et al., 2003).

#### Defects in purine metabolism and abnormal brain development: the case of tissue non-specific alkaline phosphatase (TNAP)

As highlighted in section Contribution of Purinergic Transmission to the Development of the Central Nervous System, during CNS development both extracellular nucleotides and nucleosides are directly involved in the timely and accurate modulation of neural precursor proliferation, migration, and differentiation. Among the various metabolic pathways leading to increased adenosine concentrations, a tight control of the enzymatic processes driving adenine nucleotide dephosphorylation is therefore mandatory to guarantee the correct balance between these two classes of signaling molecules.

Among enzymes involved in nucleotide catabolism, tissue non-specific alkaline phosphatase (TNAP) is peculiarly expressed in mineralizing bones, in the kidney, and in the CNS (Sebastián-Serrano et al., 2015). The main function of TNAP is to hydrolyze extracellular inorganic pyrophosphate (PPi), a potent mineralization inhibitor, thus enabling the deposition of hydroxyapatite in bones and teeth (Sebastián-Serrano et al., 2015). Additionally, TNAP is highly expressed at early stages of CNS development when proliferation of neural precursors and migration of their progeny toward their final position in the brain take place (see also Contribution of Purinergic Transmission to the Development of the Central Nervous System). Specifically, strong TNAP activity is observed at embryonic day 14 in the ventricular/subventricular zone where neural precursors are located and reside until adulthood (Langer et al., 2007). The direct role of TNAP in proliferation and differentiation processes during CNS development is still elusive, but it has now become evident that purinergic signaling plays a fundamental role in modulating proliferation, differentiation and migration capacity of neural precursors and of newborn neurons and glia cells (see Contribution of Purinergic Transmission to the Development of the Central Nervous System; Suyama et al., 2012; Boccazzi et al., 2014). Moreover, purinergic signaling is also crucially involved in axon guidance and growth and in the establishment of correct synaptic contacts, and high TNAP activity has also been documented at the stages of intensive synaptic generation and plasticity (Sebastián-Serrano et al., 2015). Based on TNAP ability to fine-tune the balance between extracellular nucleotides and nucleosides, it is therefore conceivable that this enzyme might be crucially involved in controlling the delicate phases of the building of the correct brain architecture, and in modulating the activation of specific purinergic receptors.

TNAP can also dephosphorylate pyridoxal-5′-phosphate (PLP, the active form of vitamin B6) to pyridoxal, which in turn enters the cytoplasm where it is rephosphorylated and acts as cofactor for the synthesis of enzymes involved in the metabolism of various neurotransmitters (e.g., GABA, serotonin; Amadasi et al., 2007).

Based on the above-mentioned localization and activities of TNAP, it can be speculated that defects in its expression would lead to significant pathological outcomes. In fact, hypomorphic mutations in the *ALPL* gene encoding TNAP lead to accumulation of PPi in the extracellular matrix with deficits in bone mineralization, causing hypophosphatasia, a heritable form of rickets in children or osteomalacia in adults (Table 1; Whyte, 2010). Although the exact consequences of these mutations on brain development have not been clarified yet, TNAP knock-out mice show abnormalities in myelination and synaptogenesis (Hanics et al., 2012), and subjects with hypophosphatasia suffer from seizures (Whyte, 2010), thus indicating defects in neurotransmission and/or in neurodevelopment (Fonta et al., 2015). Recent data have now started to link these defects and clinical manifestations to altered purinergic transmission, with the first demonstration of a direct involvement of an aberrant P2X7 activation in the alterations of hippocampal and cortex structure and in the development of seizures in TNAP^−/−^ mice, due to high ATP concentrations as a consequence of reduced TNAP activity (Sebastián-Serrano et al., 2016). This observation would greatly help the development of possible new pharmacological approaches to the pathology.

## Other disorders potentially due to abnormalities of purine neuromodulation during development

### Involvement of the purinergic system in brain alterations observed in down syndrome

Down Syndrome (DS) represents the most typical example of genetic disorder associated to mental retardation. Trisomy of chromosome 21 leads to the pathological overexpression of various gene products and miRNAs, but also to additional alterations spanning from the deregulation of non-coding DNA, the abnormal expression of non-HSA21 (non-*Homo sapiens* autosome 21) genes and epigenetic modifications (for review see Dierssen, 2012). These genetic abnormalities lead to a consequent dysregulation of related biochemical pathways in all tissues (Table 1; Spellman et al., 2013), but the most relevant alterations are localized in the brain, with consequent moderate to severe mental retardation (Dierssen, 2012). The most evident anatomical correlates of mental retardation in DS children and fetuses are brain hypoplasia and hypocellularity, leading to a decreased brain size (Rachidi and Lopes, 2008). Consistent with this observation, many groups have detected an impaired proliferation of neural precursors and reduction in neurogenesis in the developing neocortex, hippocampus, and cerebellum of mouse models of DS and of DS fetuses (Contestabile et al., 2007, 2009; Guidi et al., 2008). In the adult brain the above-mentioned modifications also translate in reduced spine density and impaired synaptic plasticity, paralleled by profound alterations in several neurotransmitter pathways (i.e., GABA, glutamate, serotonin etc.), with a consequent imbalance between excitatory and inhibitory neurotransmission mostly in the hippocampus (Dierssen, 2012). Additionally, amyloid precursor protein (APP) is triplicated in DS and it is believed to contribute to neurodevelopmental alterations and to trigger the development of Alzheimer-like pathology in DS adults (Stagni et al., 2017). As extensively reviewed elsewhere (Stagni et al., 2017), various biochemical pathways have been causally associated to the impairment of neurogenesis and the consequent shift in cell destiny leading to reduced neurons and increased percentage of astrocytes.

**Figure 4:**
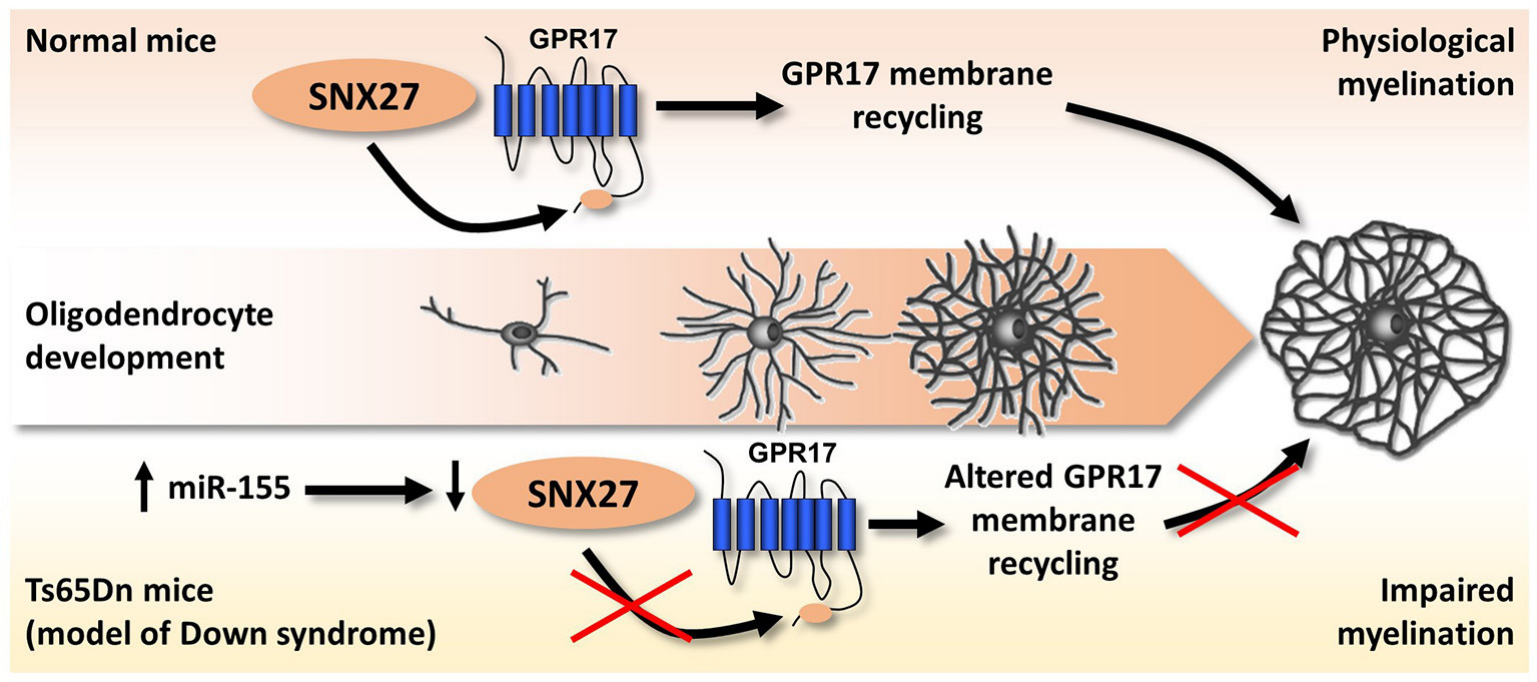
Altered SNX27/GPR17 interaction and impaired myelination in a mouse model of DS. During physiological oligodendrocyte maturation, GPR17 must be downregulated at a specific step of oligodendrocyte differentiation to allow the transition from mature to fully myelinating cells. SNX27 promotes and controls oligodendrocyte maturation, by guiding the membrane recycling and degradation of GPR17 receptor through the binding to a type I PDZ-binding motif located at its C-terminus. In the brains of Ts65Dn mice (an animal model of DS), trisomic miR-155 leads to SNX27 degradation, which in turn dysregulates GPR17 membrane expression leading to its precocious downregulation. We hypothesize that this event is crucially related to the altered pattern of myelination observed in Ts65Dn mouse brains, with reduced expression of myelin proteins, which could significantly affect cognitive functions. See text and Meraviglia et al. (2016) for details.

The first, and up to now the only available, hint of altered purinergic signaling in DS came indirectly from the observation that lack of sortin nexin 27 (SNX27), a PDZ-containing protein of the endosome-associated retromer complex controlling the trafficking of several proteins (Carlton et al., 2005), leads to severe impairment of brain functions, including cognitive manifestations mimicking those observed in DS (Wang et al., 2013, 2014). Additionally, SNX27 is down-regulated in Ts65Dn mice, the most common mouse model of DS, as a consequence of the hyperexpression of miR-155 which is physiologically responsible for its degradation (Wang et al., 2013, 2014). Our research group got interested in SNX27 while searching for the biochemical pathways responsible for the membrane-to-cytosol recycling or intracellular degradation of the G protein-coupled P2Y-like receptor GPR17. GPR17 responds to both extracellular uracil nucleotides (UDP, UDP-glucose) and cysteinyl-leukotrienes (Ciana et al., 2006). GPR17 is highly expressed by cells of the oligodendrocyte lineage, specifically during the transition from OPCs to immature oligodendrocytes. After this stage, GPR17 expression must be down-regulated to promote the generation of fully myelinating mature cells (Lecca et al., 2008; Chen et al., 2009; Boda et al., 2011; Ceruti et al., 2011; Fumagalli et al., 2011, 2015). In agreement with these findings, forced overexpression of GPR17 during late stages of differentiation leads to defective myelination *in vitro* and *in vivo*, whereas *in vivo* GPR17 knock-out accelerates oligodendrocyte myelination (Chen et al., 2009). Based on these data, we evaluated the possible involvement of SNX27 in the fine-tuned regulation of GPR17 membrane expression, and demonstrated that the endocytic trafficking of the receptor is mediated by the interaction of a type I PDZ-binding motif located at its C-terminus with SNX27. Additionally, SNX27 knock-down *in vitro* reduced GPR17 plasma membrane recycling in differentiating oligodendrocytes while fostering their terminal maturation (Meraviglia et al., 2016). When analyzing the brains of Ts65Dn mice, we observed that trisomy-linked down-regulation of SNX27 was paralleled by decreased GPR17 expression and increased number of mature oligodendrocytes, which, however, fail in reaching full maturation, eventually leading to brain hypomyelination (Meraviglia et al., 2016; Figure 4).

We therefore speculate that altered GPR17 signaling and oligodendrocyte maturation, with consequent defective axon myelination, could contribute to the overall brain pathological phenotype of DS, both in terms of brain structure and functions.

### New perspectives for the identification of causative genes involved in mental retardation: the example of PANX1

The availability of innovative and automated strategies to evaluate the presence of gene variants and mutations is accelerating our comprehension of the genetic alterations at the basis of rare or sporadic pathological phenotypes, such as some forms of mental retardation. The discovery of the first patient bearing a homozygous Pannexin 1 (Panx1) loss-of-function gene variant associated with multisystem dysfunction and intellectual disability has been recently published (Shao et al., 2016). Panx1 is a membrane channel allowing the passage of ions and small molecules, among which ATP is one of the most common (Boyce and Swayne, 2017). In the CNS, Panx1 is highly expressed by developing and mature neurons, and contributes to brain development, maturation, and to synaptic plasticity (Wicki-Stordeur et al., 2016). Thus, reduced ATP release in the presence of mutated Panx1 could lead to defective purinergic transmission which in turn could be responsible for alterations in brain structures and mental retardation. It is worth mentioning that a close relationship has been demonstrated between Panx1 and the P2X7 receptor subtype, which is crucially involved in brain development (see Contribution of Purinergic Transmission to the Development of the Central Nervous System). Data demonstrate that the two proteins can physically interact (Boyce and Swayne, 2017) and that ATP released through Panx1 in the microenvironment of P2X7 receptor ultimately leads to its activation. Defects in this signaling pathway can therefore have profound impact on brain development during embryonic and fetal life and on intellectual functions after birth.

### Maternal immune activation and neurodevelopmental abnormalities: the role of purinergic signaling

It is now increasingly recognized that inflammation is a crucial contributor to an altered fetal brain development (Hagberg et al., 2015). In particular, epidemiological studies have shown that maternal infection is an important environmental risk factor for neurodevelopmental disorders (Boksa, 2010). In preclinical animal models, perinatal infection has been indeed reported to cause maternal immune activation (also called mIA) that is associated with later appearance of autism spectrum disorder, and schizophrenia by altering fetal brain development at critical periods of pregnancy (Knuesel et al., 2014; Estes and McAllister, 2016; Table 1).

Although the initial prenatal insult alone may not be enough for clinical manifestation in human, many studies have focused on animal models of maternal infection to investigate the causal chain of events linking immune exposure and genetic predisposition, to neurodevelopmental abnormalities, as extensively reviewed elsewhere (Knuesel et al., 2014). Maternal infection is mainly mimicked by exposing pregnant rats to either LPS, or the inflammatory viral mimetic polyinosinic:polycytidylic acid (poly I:C) in the gestational period or by direct intrauterin administration of LPS (Cai et al., 2000; Bell and Hallenbeck, 2002). Immune mediators (cytokines and chemokines), reactive oxygen or nitrogen species, excitotoxicity, mitochondrial impairment, and altered vascular integrity have been described to be critical contributors to abnormal brain development in the offspring after maternal immune activation (Hagberg et al., 2015). In particular, microglial priming and elevation of maternal pro-inflammatory cytokines (i.e., TNF-α, IL-6, IL1-β) have been casually linked to dysregulation of fundamental neurodevelopment programs (Vargas et al., 2005; Morgan et al., 2010; Upthegrove et al., 2014). In this respect, members of the nucleotide-binding domain-like receptor (NLR) protein family act as inflammasome sensors, and mediate the translation of maternal immune activation to pathologically relevant neurodevelopmental abnormalities. Inflammasomes critically control different aspects of innate immunity, including the release of pro-inflammatory cytokines that induce and sustain the inflammatory response (Latz et al., 2013). The NLRP3 inflammasome is one of the major signaling routes that mediates the shift from innate immune activation to inflammation in response to different pathogenic or endogenous danger signals, including ATP (de Torre-Minguela et al., 2017). ATP-dependent stimulation of P2X7 receptors indeed promotes inflammasome activation, leading to caspase-1 cleavage and release of mature IL1-β (Ferrari et al., 2006; Di Virgilio et al., 2017). In line with this, recent data have demonstrated that both genetic deletion and the pharmacological inhibition of P2X7 receptors mitigate schizophrenia-like behavioral changes in a phencyclidine-induced rodent model of schizophrenia (Koványi et al., 2016).

Another study has revealed the critical role of P2X7 receptor in affecting astrocyte-neuron cross-talk after LPS prenatal exposure. The infection during gestation triggers the release of ATP from astrocytes via Cx43 and Panx1 unopposed channel, resulting in increased neuronal death mediated by P2X7 receptors and Panx1 channels (Avendano et al., 2015).

Maternal inflammation induced by perinatal infections can also result in preterm infants (Hagberg et al., 2012). Diffuse perinatal white matter injuries, including periventricular leukomalacia caused by ischemic damage, are the most common type of brain injury in preterm infants. Pathological demyelination and impaired oligodendrocyte maturation are known to cause cerebral palsy, and cognitive, behavioral, and sensory deficits as well as psychological problems later in life (van Tilborg et al., 2016). As detailed in section Toward a Purinergic-Based Therapy for Congenital Neurodevelopmental Disorders?, administration of UDP-glucose, acting on different purinergic receptors, has proven successful in improving the survival of newly generated oligodendrocytes in neonatal rats with ischemic periventricular leukomalacia (Mao et al., 2012; Li et al., 2015; Table 2).

**Table 2:** Purine- and pyrimidine-based pharmacological approaches to modulate congenital neurodevelopmental disorders and their functional outcomes.

**Neurodevelopmental disorder**	**Approach**	**Target receptor(s)**	**Effects**	**References**
Autism spectrum disorder	Suramin (SAT-1 translational pilot study)	P2 receptors	↑ language and social interactions ↓ repetitive behaviors	Naviaux et al., 2017
	Ketogenic diet	Mainly A_1_ receptors (increased activity)	↑ cognition, mood, behavior and social life	Masino et al., 2011b, 2013
HPRT deficiency	SAM	None	↓ self-injurious behavior	Chen et al., 2014
Ischemic periventricular leukomalacia	UDP-glucose	Activation of P2Y_14_ and GPR17 receptors	↑ proliferation and differentiation to mature oligodendrocytes of glial progenitors in the subventricular zone	Mao et al., 2012; Li et al., 2015
PRPS1-related disorders (including Arts syndrome)	SAM	None	↓ neurological symptoms	de Brouwer et al., 2010
Schizophrenia	Strategies to upregulate extracellular adenosine (see text)	Mainly A_1_ receptors (increased activity)	↓ psychotic symptoms	Shen et al., 2012
	JNJ-47965567	Block of P2X7 receptor	↑ of social interactions (phencyclidine-induced schizophrenia)	Koványi et al., 2016
UMPS deficiency	UMP and CMP administration	None	↓ neurological and non-neurological symptoms	Nyhan, 2005

Interestingly, another approach targeting purinergic receptors with suramin has been reported to correct the autism spectrum disorder-like phenotype and to restore normal social behavior in a maternal immune activation murine model of autism-like behaviors using polyI:C exposure, and in the Fragile X (*Fmr1* knockout) model (Naviaux et al., 2013, 2015; Table 2). These effects have been ascribed to the capability of the anti-purinergic treatment to normalize the expression of two purinergic receptors (P2Y_2_ and P2X7) and the phosphorylation of ERK1, ERK2, and CAMKII, all of which modulate purinergic signaling. In addition, suramin has been reported to correct the concentrations of some altered purine metabolites (9 out of 11, including ATP and allantoin) in the plasma of mice subjected to maternal immune activation (Naviaux et al., 2013, 2014, 2015). Metabolomic studies have also revealed similar alterations in the purine and pyrimidine metabolite profile in human biofluids (urines and plasma) of children suffering from autism spectrum disorder (Gevi et al., 2016). Moreover, purinergic signaling in the brain has been identified as one of the top-regulated gene expression pathways correlated with abnormal behaviors in children with autism spectrum disorder suggesting a key role of purines in driving a cell danger response in these disorders (Ginsberg et al., 2012).

Although the molecular basis of the altered purine metabolism in murine models of autism spectrum disorder still remains to be elucidated, these data suggest that metabolic pathways that synthesize and catabolize purines are critical regulatory elements in autism spectrum disorder, in accordance with their increasingly recognized role in promoting other neurodevelopmental and behavioral abnormalities (Micheli et al., 2011, see sections Defects in Nucleotide Metabolism and Toward a Purinergic-Based Therapy for Congenital Neurodevelopmental Disorders?).

## The adenosine dysfunction hypothesis in neurodevelopment

Interestingly, as elegantly summarized in a previously published review (Boison et al., 2012), it has been proposed that dysfunctions in normal adenosine homeostasis during critical early brain development may have important consequences on the formation of neuronal circuitries, thus contributing to the neurodevelopment alterations at the basis of schizophrenia (Lara and Souza, 2000; Lara et al., 2006). In particular, abnormal adenosine levels (consequent to brain insults, such as hypoxia, seizures, infections, and trauma, eventually leading to ATP breakdown or due to altered control exerted by adenosine kinase; Cunha, 2001; Dunwiddie and Masino, 2001; Boison et al., 2010) have been described to induce primary brain changes. In line with these observations, administration of an A_1_ adenosine receptor agonist immediately after birth leads to ventricular enlargement and widespread gray and white matter alterations (Turner et al., 2002), paralleled by a reduction in A_1_ receptor density. Furthermore, magnetic resonance imaging (MRI) studies in schizophrenic patients have revealed diffuse gray and white matter changes (Davis et al., 2003), that could be also related to early adenosine alterations. Interestingly, in the immature brain adenosine in mostly toxic to axons, which is in agreement with high neuronal density and reduced arborization in schizophrenic brain (Davis et al., 2003). These events would lead to deficit of adenosine-mediated inhibitory actions due to a partial loss of A_1_ receptors, which has been proposed to increase brain vulnerability to damage in the adulthood. In fact, it results in increased basal dopaminergic activity, due to attenuation of the tonic inhibition of dopamine release, leading to positive symptoms of the pathology (e.g., hallucinations), and in augmented vulnerability to excitotoxic glutamate in mature brain. On these bases, pharmacological treatments enhancing adenosine activity could be effective for symptoms control in schizophrenia (Shen et al., 2012; see section Toward a Purinergic-Based Therapy for Congenital Neurodevelopmental Disorders?).

Of note, several studies have also shown that adenosine plays a key role in reducing multiple behavioral symptoms of autism (Tanimura et al., 2010; Masino et al., 2011a), based on the well-established role of this purine nucleoside as anti-convulsant, sleep-promoter and anxiolytic (Ribeiro et al., 2002; see section Toward a Purinergic-Based Therapy for Congenital Neurodevelopmental Disorders? and Table 2).

## Toward a purinergic-based therapy for congenital neurodevelopmental disorders?

Overall, the above-mentioned evidence suggests that purinergic signaling is directly involved in many neurodevelopmental alterations that eventually lead to severe congenital disorders. In the case of exclusively genetic pathologies, the ideal strategy would be to fully restore the deficits by gene therapy. This has been successfully obtained for children affected by ADA-SCID (see section Defects in Purine *De Novo* Biosynthesis and Catabolism), by unconditioned hematopoietic stem cell transplant (Ferrua and Aiuti, 2017), due to the predominant peripheral manifestations of the pathology. To date, the correction of neurodevelopment defects through genetic *in utero* manipulation of defective pathways is still a matter of debate, also due to significant ethical issues. Thus, when modifications in enzyme and receptor activity are involved, acting pharmacologically after birth to restore defective functions can represent an innovative and feasible strategy to alleviate the symptoms.

One possibility is the dietary administration of the end-products of defective pathways. Concerning diseases linked to altered nucleotide and nucleoside metabolism (see section Neurodevelopmental Disorders Associated to Alterations in Purine and Pyrimidine Metabolism), depletion of the purine nucleotide pools observed in patients completely lacking PRPS1 or having some residual enzyme activity induces a significant ATP starvation, that results in a strong energy impairment in neurons. Ideally, direct brain administration of adenosine would be the best therapeutic strategy, as it is efficiently salvaged to corresponding nucleotides by adenosine kinase. Unfortunately, as already mentioned (see section Neurodevelopmental Diseases and Pyrimidine Metabolism), dietary adenosine itself is not absorbed by the intestine, but S-adenosylmethionine (SAM) can function as cargo to cross both the gut and the blood-brain barriers; accordingly, it has been already successfully used to treat some cases of Arts syndrome (see section Defects in Purine *de Novo* Biosynthesis and Catabolism; de Brouwer et al., 2010). Moreover, SAM has been demonstrated to reduce self-injurious behavior in children with HPRT deficiency (Chen et al., 2014). The main risk of SAM administration is the possible generation of homocysteine with consequent vascular toxicity. Thus, its extensive use in clinics should be carefully evaluated.

UMP and CMP administration was reported to have a therapeutic effect in subjects with UMPS deficiency, likely due to dephosphorylation to their respective nucleosides, able to cross the plasma membrane. Uridine supplementation was also shown to provide a good source for salvage to UMP, displaying remarkable effects on both neurological and non-neurological symptoms and even complete remission in patients with pyrimidine deficiency (Nyhan, 2005). Conversely, uracil was ineffective.

Based on the known role of purines in promoting myelination (see section Contribution of Purinergic Transmission to the development of the Central Nervous System), the presence of congenital white matter abnormalities is suggestive of alterations of specific purinergic signaling pathways. Of note, the long-term prognosis of neonatal rats with cerebral white matter injury due to ischemic periventricular leukomalacia (see section Maternal Immune Activation and Neurodevelopmental Abnormalities: the Role of Purinergic Signaling) was significantly improved by the intraperitoneal injection of UDP-glucose, an endogenous agonist acting on different purinergic receptor subtypes, including GPR17 receptor (a key determinant of oligodendrocyte maturation and differentiation; see section Involvement of the Purinergic System in Brain Alterations Observed in Down Syndrome). UDP-glucose stimulated the proliferation of glial progenitor cells derived from both the ventricular/subventricular zone and white matter, promoted their differentiation into mature oligodendrocytes, and raised the survival rate of newly generated glial cells (Mao et al., 2012; Li et al., 2015). Based on the observed dysfunctions in the pattern of myelination accompanied by reduced GPR17 expression in brains from a mouse model of DS (see section Involvement of the Purinergic System in Brain Alterations Observed in Down Syndrome), it can be speculated that the pharmacological manipulation of this receptor could prove effective in reducing DS-linked intellectual disabilities.

Autism spectrum disorder is currently lacking pharmacological approaches, also due to the wide range of associated symptoms and clinical manifestations. Based on the promising findings on a mouse model of the disease (see section Maternal Immune Activation and Neurodevelopmental Abnormalities: the Role of Purinergic Signaling), a recent small, phase I/II, randomized clinical trial has been carried out to examine the safety and activity of a single intravenous low-dose of suramin in children with autism spectrum disorder D. The Suramin Autism Treatment-1 (called SAT-1) was a double-blind, placebo controlled, translational pilot study that showed improvements in language, and social interaction and decreased repetitive behaviors (Naviaux et al., 2017). Despite some limitations of this study, included its small size and the suboptimal timing of the outcome measurements, and the need to confirm the results in a larger number of children (Naviaux et al., 2017), the SAT-1 trial highlighted suramin as an encouraging innovative approach for autism spectrum disorder therapy.

Furthermore, results from diverse clinical trials have shown that a ketogenic diet (a high fat, low carbohydrate, adequate protein formula which promotes the use of ketones, rather than glucose, for energy production) can improve cognition, mood, behavior, and social life in autism spectrum disorder patients. Interestingly, these positive effects have been also ascribed to an augmented action of adenosine at A_1_ receptors, leading to opening of K^+^ channels and membrane hyperpolarization/reduced excitability (Kawamura et al., 2010; Masino et al., 2011b). Currently, two independent studies on the effects of an intermittent ketogenic diet on the behavior of children with autism spectrum disorder have been performed, and results indicated that the most significant improvements were noticed in patients showing only mild autistic behavior (Herbert and Buckley, 2013; Masino et al., 2013).

Likewise, patients affected by schizophrenia would benefit from increased adenosine receptor activity (see section The Adenosine Dysfunction Hypothesis in Neurodevelopment). Promising strategies to upregulate extracellular adenosine are represented by the inhibition of adenosine kinase (e.g., with direct inhibitors, ketogenic diet that downregulate the enzyme, or allopurinol and dipyridamole, which augment extracellular adenosine by inhibiting its degradation and reuptake) and by the use of brain implants of adenosine-releasing cells (Shen et al., 2012).

It is therefore evident that encouraging attempts to pharmacologically target the purinergic system in disorders associated to neurodevelopment have been already performed (see Table 2 for summary). Future research should now move to different directions: (i) to implement the knowledge on the physiological role played by purines/pyrimidines in controlling and promoting CNS development (see section Contribution of Purinergic Transmission to the Development of the Central Nervous System); (ii) to highlight the contribution to altered neurodevelopmental stages of dysfunction/mutations of specific enzymes or receptor subtypes and of their cross-talk [e.g., the TNAP enzyme and the P2X7-Pax1 complex; see sections Defects in Purine Metabolism and Abnormal Brain Development: the Case of Tissue Non Specific Alkaline Phosphatase (TNAP) and New Perspectives for the Identification of Causative Genes Involved in Mental Retardation: the Example of PANX1]; (iii) to foster the design and synthesis of more selective, potent, and brain permeable ligands, and their evaluation in pre-clinical animal models of the diseases. The goal to cure congenital neurodevelopmental disorders through the purinergic system will be only achieved thanks to the collaborations of different and complementary scientific expertizes, including neuroscience, neurobiology, genetics, physiology, neuropharmacology, and medicinal chemistry.

## Author contributions

All authors listed have made a substantial, direct and intellectual contribution to the work, and approved it for publication.

### Conflict of interest statement

The authors declare that the research was conducted in the absence of any commercial or financial relationships that could be construed as a potential conflict of interest.
